# Prevalence of Sensitive Terms in Clinical Notes Using Natural Language Processing Techniques: Observational Study

**DOI:** 10.2196/38482

**Published:** 2022-06-10

**Authors:** Jennifer Lee, Samuel Yang, Cynthia Holland-Hall, Emre Sezgin, Manjot Gill, Simon Linwood, Yungui Huang, Jeffrey Hoffman

**Affiliations:** 1 Nationwide Children's Hospital Columbus, OH United States; 2 The Ohio State University College of Medicine Columbus, OH United States

**Keywords:** adolescent, child, privacy, patient portals, natural language processing, eHealth

## Abstract

**Background:**

With the increased sharing of electronic health information as required by the US 21st Century Cures Act, there is an increased risk of breaching patient, parent, or guardian confidentiality. The prevalence of sensitive terms in clinical notes is not known.

**Objective:**

The aim of this study is to define sensitive terms that represent the documentation of content that may be private and determine the prevalence and characteristics of provider notes that contain sensitive terms.

**Methods:**

Using keyword expansion, we defined a list of 781 sensitive terms. We searched all provider history and physical, progress, consult, and discharge summary notes for patients aged 0-21 years written between January 1, 2019, and December 31, 2019, for a direct string match of sensitive terms. We calculated the prevalence of notes with sensitive terms and characterized clinical encounters and patient characteristics.

**Results:**

Sensitive terms were present in notes from every clinical context in all pediatric ages. Terms related to the mental health category were most used overall (254,975/1,338,297, 19.5%), but terms related to substance abuse and reproductive health were most common in patients aged 0-3 years. History and physical notes (19,854/34,771, 57.1%) and ambulatory progress notes (265,302/563,273, 47.1%) were most likely to include sensitive terms. The highest prevalence of notes with sensitive terms was found in pain management (950/1112, 85.4%) and child abuse (1092/1282, 85.2%) clinics.

**Conclusions:**

Notes containing sensitive terms are not limited to adolescent patients, specific note types, or certain specialties. Recognition of sensitive terms across all ages and clinical settings complicates efforts to protect patient and caregiver privacy in the era of information-blocking regulations.

## Introduction

With the increased sharing of electronic health information (EHI) through patient portals as the result of the US 21st Century Cures Act information blocking regulations, there is an increased risk of sharing sensitive information with the wrong person [1]. For pediatric patients and their parents or guardians, there are two major types of risk. The first is disclosure of sensitive information to the patient, which a parent or guardian wants to remain private. In a recent position statement, the Society for Adolescent Health and Medicine supports the parent’s right to withhold “certain family information” such as HIV status, substance use disorders, or consanguinity with the child [2]. The second type of risk is disclosure of sensitive information that the child or patient desires (and may be legally entitled) to withhold from a parent or guardian such as documentation of certain types of reproductive health care, mental health care, or substance abuse treatment [3-5].

Institutions rely on providers to manually flag notes that contain sensitive information [6,7]. At one pediatric institution, Parsons et al [7] manually reviewed notes flagged as sensitive (which accounted for only 2.3% of the total note volume) and found that 16% of them did not have discernable sensitive information. This aligns with the findings of prior work that providers often do not have awareness of the relevant adolescent consent laws in their state [8].

The percentage of notes flagged as containing sensitive information should be higher than indicated in the study conducted by Parsons et al [7]. In newborn patient notes, it is routine practice to document intrauterine drug use or exposure to infectious diseases such as maternal HIV. Bright Futures Guidelines from the American Academy of Pediatrics recommends providers perform psychosocial screening on patients of all ages, and substance use and sexual health screening in all adolescent patients [9,10]. In a recent survey of 3533 high school–aged adolescents, 71% confirmed that providers interviewed them without a parent present [11], in which case it should lead to the documentation of that private interview. Because current electronic health record (EHR) systems are not able to automatically identify sensitive information in clinical notes, the overall prevalence of sensitive information documented in pediatric clinical notes cannot be easily ascertained. The aim of this study is to define a keyword set of sensitive terms and characterize the prevalence of provider notes that contain sensitive terms across different clinical settings.

## Methods

### Setting

This study was a single-center retrospective review of provider notes in patients aged 0-21 years from January 1, 2019, to December 31, 2019, at an urban, academic, not-for-profit, freestanding children’s hospital with over 50 subspecialties and 1.6 million patient visits annually. The note types included were history and physical (H&P) notes, progress notes (inpatient, emergency care, and ambulatory), consultation notes, and discharge summaries authored by physicians (residents, fellows, and attendings) and advanced practice providers (nurse practitioners and physician assistants) documented in the local Epic EHR (Epic Systems Corporation).

### Study Design

We used natural language processing (NLP) term expansion to create a representative list of sensitive terms or phrases in the following four categories of sensitive information as determined by local experts: substance use, mental health, reproductive health, and home environment. These categories were created based on prior work to categorize confidential content to incorporate the most common types of sensitive content that may warrant protection from disclosure [7]. Mental health and substance use disorder records are subject to additional Health Insurance Portability and Accountability Act privacy protections [12,13]. Similarly, adolescents may consent to several elements of their own reproductive health care without parental involvement (though specific elements of care vary by state) [14-17]. Home environment includes topics the disclosure of which may place a child in danger in the home, such as parental discord or domestic violence [18]. Similar term expansion methods have been used before, such as for identification of smoking status in free-text data [19,20].

For each category, subject matter experts (SY, CH, and JL) identified 5 to 10 representative terms. We then employed a locally developed NLP tool dubbed DeepSuggest for term expansion. DeepSuggest was trained on approximately 93 million clinical notes in the EHR data set. For each term, DeepSuggest identified 60 additional potentially related terms or phrases, as well as common abbreviations and misspellings [21]. For example, for the term “alcohol,” DeepSuggest produced related terms such as “etoh” (abbreviation), “drug” (related term), and “alchol” (misspelling).

**Figure 1:**
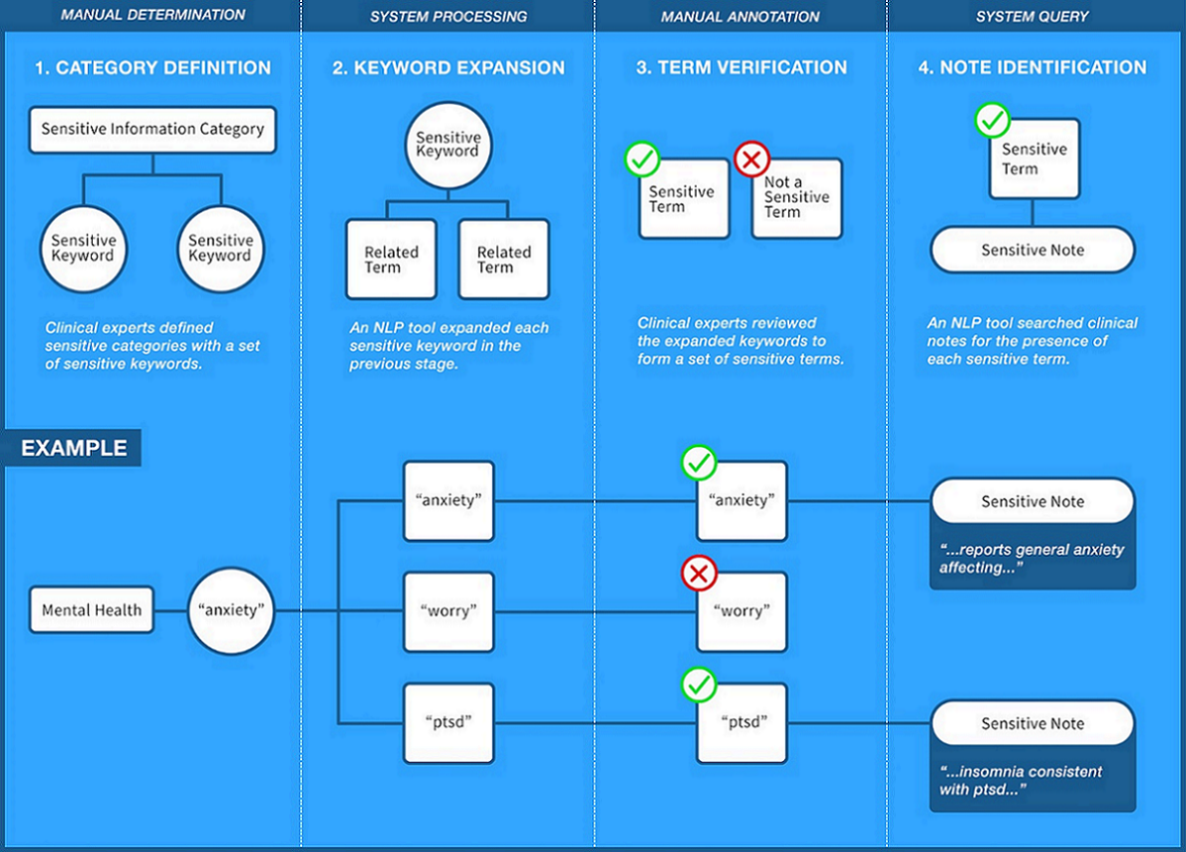
Combining natural language processing (NLP) and expert definitions to identify sensitive notes in the electronic health record. Overview of sensitive term identification protocol. Four categories and sensitive keywords representative for each category were identified by local subject matter experts. A natural language processing tool trained on the entire cohort of notes at the organization was used for keyword expansion. Each sensitive keyword was expanded to 60 potentially related terms. Each related term was manually annotated as a "sensitive" or "not sensitive" term by board-certified pediatricians and adolescent medicine specialists. Exact string word matching was used to determine if a sensitive term was documented in a clinical note; ptsd: posttraumatic stress disorder.

Two subject matter experts (JL and CH) manually annotated each term provided by DeepSuggest as either “sensitive” or “not sensitive” (Figure 1). A term was defined as “sensitive” if its presence in a clinical note could indicate documentation of a topic that might reflect sensitive information. For example, as shown in Figure 1, the initial term “anxiety” is expanded by DeepSuggest to include terms such as “worry” and “ptsd.” “Worry” is related to “anxiety” but was deemed not likely to represent sensitive content and was thus not included in the final vocabulary. Disagreements between the 2 subject matter experts was resolved through discussion.

We used direct string matching to query for the presence of any sensitive term or phrase in the selected note types. Notes documented in the EHR could contain free text, dictated or transcribed text, templated text, or dynamic links that insert discrete data from elsewhere in the EHR (eg, family history, tobacco use screening, or problem list). However, because the notes are saved as plain text, we were able to use direct string matching to screen for the presence of a term or phrase regardless of how each portion of the note was populated. Moreover, as exact string matching was used, manual review to confirm the accuracy or recall of the search parameters was unnecessary. Parsons et al [7] included the presence of psychiatric or substance use screening questions regardless of positive or negative status as confidential information. Similarly, we determined the presence of a sensitive term, regardless of negation status, as sensitive information. Figure 1 describes the study design.

Clinical notes identified by the search were stratified by note type, author type, and patient age. Because most patient encounters were ambulatory encounters, these notes were also stratified by specialty.

### Analysis

Descriptive analysis was performed for all notes by patient cohort, encounter type, and author provider type. We identified the top 10 frequently occurring sensitive terms by category and compared the prevalence of clinical notes with sensitive terms among note types by age using the Fisher exact test (*P*<.05). We used the Cohen kappa to quantify interrater agreement for sensitive term identification. We then determined the prevalence of clinical notes with sensitive terms written in the ambulatory setting and compared them by clinical specialty, also using the Fisher exact test (*P*<.05).

### Ethics Approval

The study was approved by the Nationwide Children’s Hospital Institutional Review Board (STUDY00000611).

## Results

In the study period, there were 763,133 clinical encounters among 279,737 unique patients. In total, 70.3% (536,201/763,133) of the encounters occurred in an ambulatory setting; 20.7% (70,378/763,133) of the patients were 13 years or older. Most patients were White (151,988/279,737, 54.3%), and there was a slight male predominance (142,539/279,737, 51.0% male vs 137,180/279,737, 49.0% female). During the study period, a total of 1,338,297 notes were written by 2342 unique providers, with 501,762/1,338,297 (37.5%) notes containing at least one sensitive term (Table 1).

**Table 1:** Patient, encounter, and provider characteristics.

Populations and characteristics			Values, n (%)
**Patients (n=279,737)**			
	**Age (years)**		
		Less than 13	209,359 (74.84)
		13 to 18	59,415 (21.23)
		18 to 21	10,963 (3.92)
	**Legal sex**		
		Male	142,539 (50.95)
		Female	137,180 (49.04)
		Unknown	18 (0.01)
	**Race**		
		White	151,988 (54.33)
		Black or African American	66,995 (23.95)
		Latino or Hispanic	19,053 (6.81)
		Other or unknown	41,701 (14.91)
**Encounters**			763,133
	Ambulatory care		536,201 (70.3)
	Emergency care		188,204 (24.7)
	Inpatient care		38,728 (5.1)
**Providers (n=2342)**			
	Resident		888 (37.92)
	Attending		828 (35.35)
	Fellow		393 (16.78)
	Advanced practice provider		233 (9.94)
**Notes (n=1,338,297)**			
	Notes with sensitive terms		501,762 (37.49)

DeepSuggest expanded 27 sensitive keywords to 1620 new candidate terms. Of those 1620 terms, 478 (30%) were duplicates; 781 (68%) of the 1142 unique candidate terms were determined to be sensitive with an interrater reliability (kappa score) of 0.944. Of the 781 sensitive terms, 698 (89%) were found in the study period (supplemental Table for list of initial keywords and full list of terms are presented in Multimedia Appendix 1).

“Anxiety” was the most frequent sensitive term, with 418,766 total occurrences among 143,968 notes. “Depression” had fewer mentions than anxiety, occurring in 150,934 notes. Abbreviations such as “thc” (tetrahydrocannabinol), “cps” (child protective services), “si” (suicidal ideation), and “hiv” (human immunodeficiency virus) were among the terms most commonly found in the notes. Table 2 describes the 10 most frequent terms in each of the 4 categories.

**Table 2:** Most frequently used sensitive terms by category.

Category and term		Term frequency, n	Note frequency, n
**Substance use**			
	tobacco	190,547	119,764
	alcohol	143,945	107,871
	substance	101,183	78,997
	smoker	51,572	50,538
	cigarettes	36,444	35,443
	substance abuse	28,970	23,131
	thc^a^	21,216	14,153
	marijuana	16,625	10,985
	smoked	14,508	14,271
	cocaine	13,747	8618
**Mental health**			
	anxiety	418,766	143,968
	depression	270,661	150,934
	mood	267,706	122,293
	suicidal	224,989	72,709
	suicidal ideation	140,918	57,057
	suicide	109,123	46,463
	si^b^	66,977	35,713
	panic	52,040	32,025
	bipolar	46,729	35,539
	depressive	41,511	26,025
**Reproductive health**			
	sexual	238,310	84,710
	pregnancy	118,872	77,337
	hiv	80,306	56,072
	partner	62,456	33,155
	sexually	44,491	33,902
	sexually active	37,149	29,817
	sexual abuse	36,000	16,030
	sti^c^	33,904	22,679
	sex	30,612	21,714
	partners	23,406	13,461
**Home environment**			
	abuse	156,957	70,712
	food insecurity	21,108	14,848
	bullying	17,259	10,712
	conflict	14,997	9657
	cps^d^	13,962	9081
	weapons	12,016	9485
	abuse or neglect	11,671	6212
	emotional abuse or neglect	11,195	5784
	perpetration	11,017	5403
	ycsu^e^	10,511	6488

^a^thc: tetrahydrocannabinol.

^b^si: suicidal ideation.

^c^sti: sexually transmitted infection.

^d^cps: Child Protective Services.

^e^ycsu: Youth Christian Social Union.

**Figure 2:**
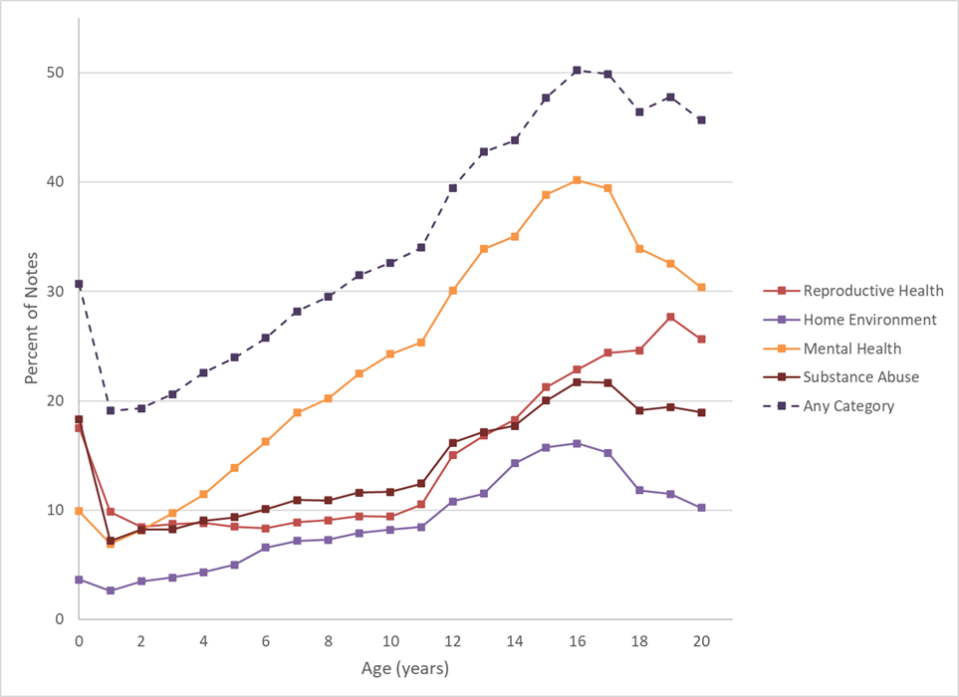
Percent of notes containing sensitive terms by age of patient and category. Line graph depicting percent of clinical notes containing at least one sensitive term over age. Sensitive terms are found in a portion of clinical notes for all patient ages. This figure demonstrates that while all categories show an upward trend during adolescent age, in the first year of life, reproductive health and substance abuse categories are the most frequently documented.

Mental health terms were documented most, occurring in 254,975 (19.5%) of notes, followed by reproductive health in 184,720 (14.2%), substance use in 184,342 (14.2%), and home environment in 95,598 (7.4%). The difference in term prevalence between mental health and the next closest category (reproductive health) was statistically significant (*P*<.05). This difference was most notable in the adolescent years. In the first year of life, substance use and reproductive health terms are more frequently documented than terms from the other two categories. Figure 2 demonstrates the prevalence of any term from different categories by age.

**Figure 3:**
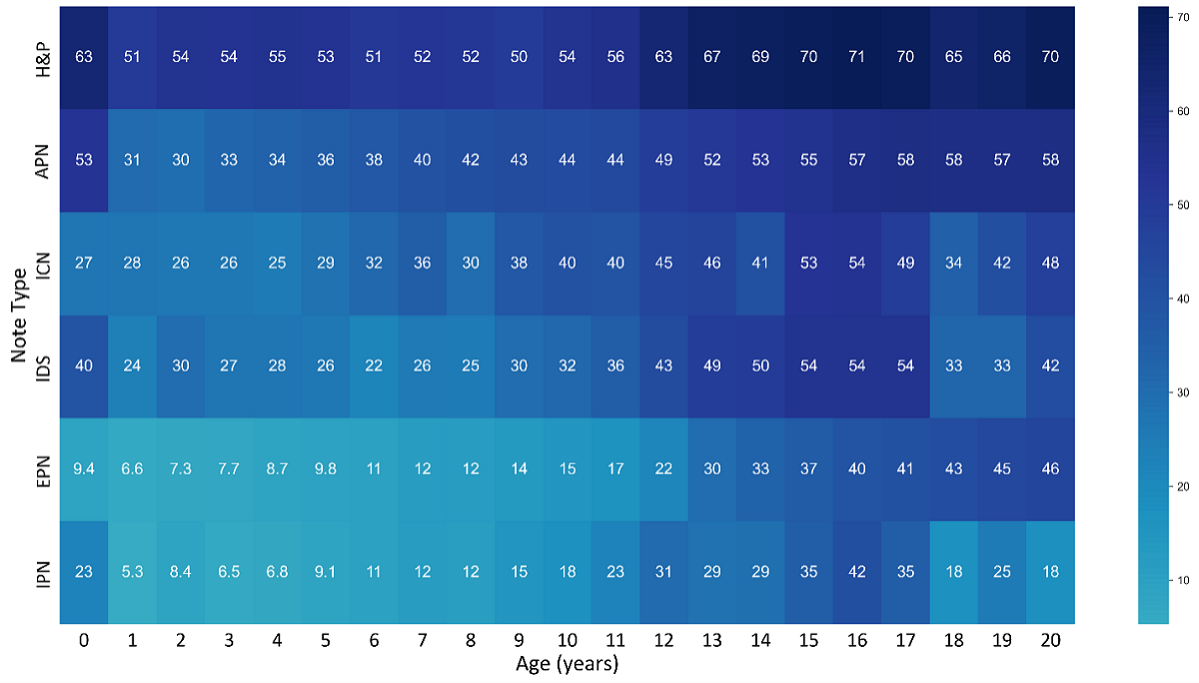
Percent of notes containing sensitive terms by age and note type. This heat map demonstrates the specific note types that contain at least one sensitive term of any category. Sensitive terms are found in a portion of all clinical note types examined in all age groups. This figure demonstrates that while all categories show an upward trend of including sensitive notes during adolescent age, the history and physical note is most likely to contain sensitive term overall. APN: ambulatory progress note; EPN: emergency care and urgent care progress note; H&P: history and physical note; ICN: inpatient consult note; IDS: inpatient discharge summary; IPN: inpatient progress note.

The prevalence of sensitive terms varied by note type. The inpatient H&P note type contained at least one sensitive term 57.1% (19,854/34,771) of the time, whereas ambulatory progress notes contained sensitive terms in 46.7% (265,302/563,273) of cases. Figure 3 shows a heat map of the percentage of notes containing a sensitive term by note type and age. The notes with the highest percentage sensitive terms are H&P notes among adolescent patients aged 12-20 years (66%-73%).

In terms of specialty, the highest prevalence of ambulatory progress notes with at least one sensitive term occurred in pain management (950/1112, 85.4%) child abuse pediatrics (1092/1282, 85.2%), obstetrics or gynecology (5701/6707, 85.0%), and behavioral health (2128/2589, 82.2%). The difference between obstetrics or gynecology and behavioral health was statistically significant (*P*<.05). Pediatric primary care had an overall prevalence of 44.0% (175,173/398,120) of notes with sensitive terms.

## Discussion

### Principal Findings

To our knowledge, our study is one of the first studies to define sensitive terms to represent categories of confidential information using NLP. In this study, sensitive terms were identified in notes from every clinical context, provider type, specialty, and in all ages included in the study cohort. Prior to the 21st Century Cures Act information blocking regulations, sharing of notes through the portal was not mandatory. Organizations voluntarily sharing notes (eg, the OpenNotes initiative) commonly prevented the release of notes in specific specialties and, in particular, among the adolescent age group to protect confidentiality [22,23]. Now, federal regulations limit the circumstances under which health care providers may withhold EHI. Moreover, our data show that sensitive terms are present diffusely across all notes in our system, making approaches that restrict notes within specific specialties or certain age groups no longer viable options.

Institutions rely on manual notation by the author [7] even though research has shown that providers may not be aware of the confidentiality laws in their state [8]. Our work indicates that there is no generalizable rule that can be applied to prevent unintended disclosure of sensitive terms in clinical notes. It is important to note that the presence of a sensitive term in a clinical note is not equivalent to an EHI that should be withheld. Instead, providers need to be cognizant of sensitive term documentation before sharing with a patient, parent, or guardian. Future work is being carried out at our organization to alert authors of the presence of sensitive terms before releasing to a portal.

We found that sensitive terms related to mental health were the most common overall, but in the first years of life, terms related to reproductive health and substance abuse were more prevalent. This is most likely due to the documentation of maternal history [24]. Disclosure of maternal history may lead to privacy violations when viewed by another legal guardian or by the patient at an older age [25].

Inpatient H&P and ambulatory progress note types have a higher prevalence of sensitive terms across all age groups. This may be due to the documentation of various screening tools used during patient encounters. For example, the American Academy of Pediatrics recommends universal psychosocial and depression screening beginning at the age of 11 years and risk assessment for alcohol and drug use during well-child exams [9,10]. The US Preventive Services Task Force recommends routinely screening adolescents for HIV starting at the age of 15 years [26,27]. To facilitate compliance with screening for billing purposes, clinical note templates often include these screening questions, along with their requisite sensitive terms, to prompt clinicians during the visit. Adolescent patients in ambulatory settings report having frequent private conversations with their provider [11]; however, in the pediatric inpatient setting, nonobservance of privacy protections is often reported [28].

The notes with highest prevalence of sensitive terms were adolescent patient H&P notes. Studies have shown adolescents would forgo care if confidentiality regarding sensitive issues was not assured [29,30]. In addition to missed care, the release of sensitive information for adolescents may constitute a breach of state or federal privacy law. Individual health systems define different types of portal access, often giving adolescent patients full or limited access [31]. Under the Health Insurance Portability and Accountability Act, parents and legal guardians are considered personal representatives of patients under 18 years (ie, minors) and are thus afforded proxy access to the patient’s EHI, including access through patient portals [16,23,31]. However, a recent work by Ip et al [32] demonstrated that parents are often active users of adolescent portal accounts, making it even more crucial that note authors recognize sensitive content in their notes and take into consideration who can see what in their patient portal.

### Clinical Implications

The presence of sensitive terms in a clinical note does not necessarily indicate that a note is to be considered confidential. However, confidential notes likely contain sensitive terms. Providers need to be educated on what information is protected by federal and state laws, and they should determine, on a case-by-case basis, which notes are not to be shared. Furthermore, patients and guardians should be informed regarding who has access to what information in a patient portal, and proxy access policies should be regularly reviewed and updated as needed. Ideally, sensitive conversations with patients or guardians should also include discussion about whether this information should be kept confidential or shared through the patient portal.

Our approach identified sensitive terms anywhere in the body of a clinical note regardless of whether it was entered manually by the provider or added to the note from discrete data sources elsewhere in the EHR, such as prior family or social history documentation. For example, if a patient’s problem list contains a sensitive term such as “prior suicide attempt” and the problem list is included in a note template, it may be added automatically to a clinical note for a visit unrelated to a sensitive condition, thus rendering the note inadvertently confidential. Similarly, copy and paste behavior can result in unrecognized inclusion of sensitive information in otherwise nonconfidential notes. For these reasons, additional work is needed to identify the source of the sensitive terms found in Figure 3.

### Policy Considerations

This study demonstrates that sensitive terms are documented in clinical notes across all ages, including an increase in mental health–related terms starting at the age of 10 years. Laws and institutional policies are often designed to protect adolescent privacy; however, there is often a lack of protection for other age groups. Current law and policies might need to be revisited in light of this research.

### Future Development

Further technological development is needed for EHRs and other health information technologies to support improved protection of patient and guardian privacy. Tools based on NLP techniques may now be possible, which could provide real-time feedback to note authors in situations where sensitive content present in clinical documentation may not otherwise be recognized and protected from inadvertent disclosure. Several challenges may be encountered when considering the implementation of similar NLP-supported tools [33]. Prior to implementation, a health institution must ensure data privacy and integrity, consider the necessities of information system infrastructure, model, and system performance, as well as performing assessment for algorithmic bias [33-35]. From a provider standpoint, as many institutions are working on reducing provider alert burden [36], they should be cautious toward implementing such tools not to increase provider alerting, which has been associated with provider burnout. As such, provider acceptance of these tools should be monitored over time [33].

### Limitations

We defined sensitive terms broadly to increase the likelihood of identifying notes that might contain information that should remain confidential. However, the presence of a sensitive term by itself does not equate to confidential content. For example, “partner” (or “partners”) is a very common term in the reproductive health category. This word could be used in phrases that indicate confidential content, such as “sexual partner,” but also in nonconfidential content such as “partners with teachers to assess behavior at school.” This highlights the need for providers to make the final determination of whether a clinical note contains confidential content.

Our list of sensitive terms is not comprehensive. DeepSuggest expands a single keyword to up to 60 potentially related terms in an unsupervised manner. However, given that less than 50% of the expanded terms were considered sensitive, expanding the potentially related term set may not improve the identification of additional sensitive terms. In future work, these sensitive term findings may be used to develop a specific algorithm to locate sensitive terms with a greater degree of precision. For instance, the deep learning algorithms have been successfully used to identify adverse events [37].

### Conclusion

Clinical notes often contain sensitive terms and thus pose a challenge in complying with new regulations that require more timely and transparent disclosure of clinical notes to patients, parents, and legal guardians. Confidential information protected by law and ethical standards should be withheld from disclosure. The presence of sensitive terms in a clinical note may indicate documentation of confidential information requiring protection from inadvertent disclosure. To the best of our knowledge, this is the first study that defines sensitive terms in this context using an iterative process of expert opinion and NLP techniques, thus allowing an approximation of the actual prevalence of sensitive terms in provider clinical notes in a pediatric population. We hope this work is the first step toward developing tools to assist providers in identifying potentially confidential information present in their clinical notes, thereby avoiding accidental disclosure to the wrong person.

## Appendix

Multimedia Appendix 1 Supplemental table of sensitive terms.

## Abbreviations

EHI: electronic health information
EHR: electronic health record
H&P: history and physical
NLP: natural language processing
